# Consumo Abusivo de Álcool em Cardiopatas: Análise de Risco pelo Comportamento de Estilo de Vida – Projeto PROSA (Projeto Saúde e Álcool)

**DOI:** 10.36660/abc.20240744

**Published:** 2025-10-09

**Authors:** Martino Martinelli-Filho, Caio Vitale Spaggiari, Roberta Vanalli Baroni, Cinthya Ibrahim Guirao Gomes, Thiago Ovanessian Hueb, Sergio Augusto Mezzalira Martins, Anísio Alexandre Andrade Pedrosa, Silvana Angelina Dório Nishioka, Sergio Freitas de Siqueira

**Affiliations:** 1 Hospital das Clínicas Faculdade de Medicina Universidade de São Paulo São Paulo SP Brasil Instituto do Coração do Hospital das Clínicas da Faculdade de Medicina da Universidade de São Paulo, São Paulo, SP – Brasil

**Keywords:** Consumo Excessivo de Bebidas Alcoólicas, Estilo de Vida, Cardiopatias

## Abstract

**Fundamento:**

A ingestão moderada de álcool parece ter um efeito protetor cardiovascular, enquanto o consumo nocivo de álcool (CNA) está associado a altas taxas de doenças crônicas e mortalidade. A quantidade de álcool ingerido tem sido associada a comportamentos de estilo de vida; no entanto, essa relação ainda não foi avaliada em pacientes com doença cardíaca (DC).

**Objetivo:**

Avaliar a associação entre o estilo de vida e o CNA em pacientes com DC.

**Métodos:**

Trata-se de um estudo observacional que incluiu um grupo de pacientes com DC e um grupo de voluntários, com o objetivo de avaliar a associação entre o CNA e o comportamento de estilo de vida utilizando um escore de estilo de vida (o escore LBS). O consumo de álcool foi avaliado por meio do teste AUDIT-C. O LBS foi desenvolvido atribuindo-se 1 ponto para cada variável — tabagismo; sobrepeso ou obesidade (índice de massa corporal ≥24,9); ansiedade; depressão; e falta de atividade física — e classificado como saudável (0/1 ponto), regular (2/3 pontos) ou não saudável (4/5 pontos). A análise estatística adotou como nível de significância o valor de p < 0,05.

**Resultados:**

O estudo incluiu 1999 pacientes com DC e 2081 voluntários. A análise de regressão, controlada por idade e sexo, revelou que os escores de estilo de vida regular e não saudável são preditores independentes do CNA em pacientes com DC (OR = 1,35, p = 0,04 e OR = 1,76, p = 0,001, respectivamente), similarmente ao observado nos voluntários. A validação temporal demonstrou boa capacidade de discriminação do modelo de regressão (ROC = 0,80). Além disso, melhorias no escore de estilo de vida (LBS) foram associadas à redução do CNA (p = 0,02).

**Conclusões:**

Este estudo demonstrou que a avaliação do risco de HAC por meio de um novo escore de estilo de vida é um preditor confiável em pacientes com DC. Também foi observado que a melhora nos comportamentos de estilo de vida pode contribuir para a redução do CNA.

## Graphical abstract

**Figura Central:**
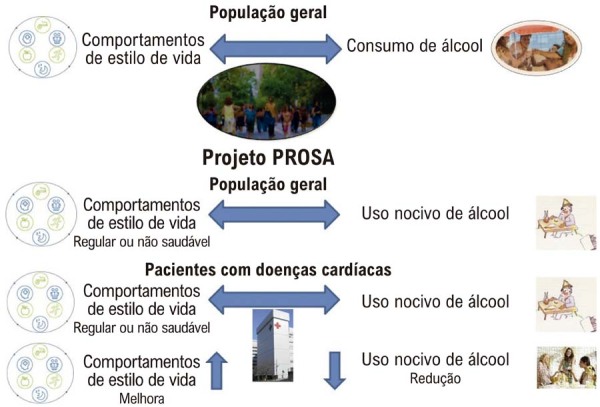
Consumo Abusivo de Álcool em Cardiopatas: Análise de Risco pelo Comportamento de Estilo de Vida – Projeto PROSA (Projeto Saúde e Álcool)

## Introdução

Diversos estudos sugerem potenciais benefícios cardiovasculares do consumo leve a moderado de álcool na população geral, indicando um efeito protetor contra doenças cardiovasculares.^1-4^ No entanto, para compreender a real relação entre consumo de álcool e desfechos em saúde, deve-se considerar diversas variáveis de confusão, o que ainda não foi avaliado em pacientes com doença cardíaca (DC).^5-7^

Nesse sentido, a adoção de um estilo de vida saudável tem sido associada à redução do risco de doenças crônicas importantes, incluindo câncer, infarto, hipertensão, acidente vascular cerebral, diabetes e distúrbios do ritmo cardíaco.^1,2^ Em contraste, o consumo nocivo de álcool (CNA) é reconhecido como um fator que contribui negativamente para os desfechos em saúde, agravando doenças crônicas e aumentando tanto a morbidade quanto a mortalidade.^8-10^ Em pacientes com DC, a correlação entre consumo de álcool e desfechos em saúde segue um padrão dose-resposta.^1 1^ Nesse contexto, o CNA pode ter consequências graves ao agravar condições pré-existentes e representar ameaças significativas à saúde dos pacientes.^10-12^

As iniciativas globais de saúde – exemplificadas pela Organização Mundial da Saúde (OMS) – têm buscado promover pesquisas voltadas à compreensão e redução da prevalência do CNA em diversas populações.^1 3^ O principal desafio permanece em estabelecer uma relação causal entre o consumo de álcool e o início ou a progressão de doenças crônicas.

Nesse contexto, esforços têm sido direcionados à avaliação dos riscos potenciais do consumo de álcool, especialmente em indivíduos com condições de saúde pré-existentes, associando esses riscos a comportamentos de estilo de vida. O tabagismo, por exemplo, não apenas apresenta uma forte associação com a doença pulmonar obstrutiva crônica e o câncer de pulmão, como também é reconhecido por sua correlação com o CNA.^14-18^

O desenvolvimento de uma ferramenta de avaliação do estilo de vida específica para a população, o *Lifestyle Behavior Score* (LBS), é fundamental para identificar fatores associados aos padrões de consumo de álcool e permitir análises estatísticas robustas adaptadas a populações específicas. Assim, nossa hipótese propõe uma forte associação entre um LBS não saudável e o CNA, particularmente em pacientes com DC. Portanto, este estudo tem como objetivo avaliar a associação entre o LBS e o CNA em indivíduos com e sem DC.

## Métodos

Este é um estudo prospectivo que incluiu duas coortes distintas: pacientes com DC e voluntários, categorizados de acordo com o padrão de consumo de álcool.

### População do estudo

A coorte de pacientes com DC incluiu pacientes clinicamente estáveis, diagnosticados com qualquer forma de cardiomiopatia, em seguimento ambulatorial no Instituto do Coração (InCor), no Hospital das Clínicas da Faculdade de Medicina da Universidade de São Paulo (HCFMUSP). A coorte de voluntários foi recrutada entre familiares ou cuidadores dos pacientes com DC, e entre funcionários do hospital. Ambas as coortes incluíram indivíduos de qualquer sexo, gênero ou raça, com 18 anos de idade ou mais. Consentimento informado foi obtido de todos os participantes, e o protocolo do estudo foi aprovado pelo Comitê de Ética do InCor-HCFMUSP (CAAE 61741916.0.0000.0068 número do protocolo de aprovação 1.846.682, 02 de dezembro, 2016).

### Consumo de álcool, padrão do consumo e escore de comportamento de estilo de vida

Para avaliar o consumo de álcool, aplicamos o *Alcohol Use Disorders Identification Test* (C) (Material Suplementar).^19-21^ No entanto, o AUDIT-C não especifica o tipo de bebida alcoólica consumida e, por isso, utilizamos o questionário institucional Q-PROSA (Material Suplementar) para avaliar com maior precisão o tipo de bebida preferido – cerveja, destilados e/ou vinho – bem como as quantidades consumidas. Os participantes foram caracterizados de acordo com a quantidade de álcool ingerida. O CNA foi classificado como >28g de etanol por dia para homens e >14g de etanol por dia para mulheres, ou como consumo episódico excessivo (*binge*) conforme o escore do AUDIT-C.

Para avaliar o comportamento relacionado ao estilo de vida, diversos fatores foram avaliados por meio de questionários padronizados. Os níveis de depressão e ansiedade foram avaliados utilizando a Escala Hospitalar de Ansiedade e Depressão (HADS, *Hospital Anxiety and Depression Scale*),^2 2^ e os níveis de atividade física foram mensurados usando o Questionário Internacional de Atividade Física (IPAQ, *International Physical Activity Questionnaire*).^2 3^ O status de tabagismo, a escolaridade e a renda foram avaliados por meio de perguntas objetivas. Além disso, medidas de peso e altura foram obtidas de todos os participantes. Todos os questionários utilizados eram versões validadas para a língua portuguesa do Brasil.

**Figura 1:**
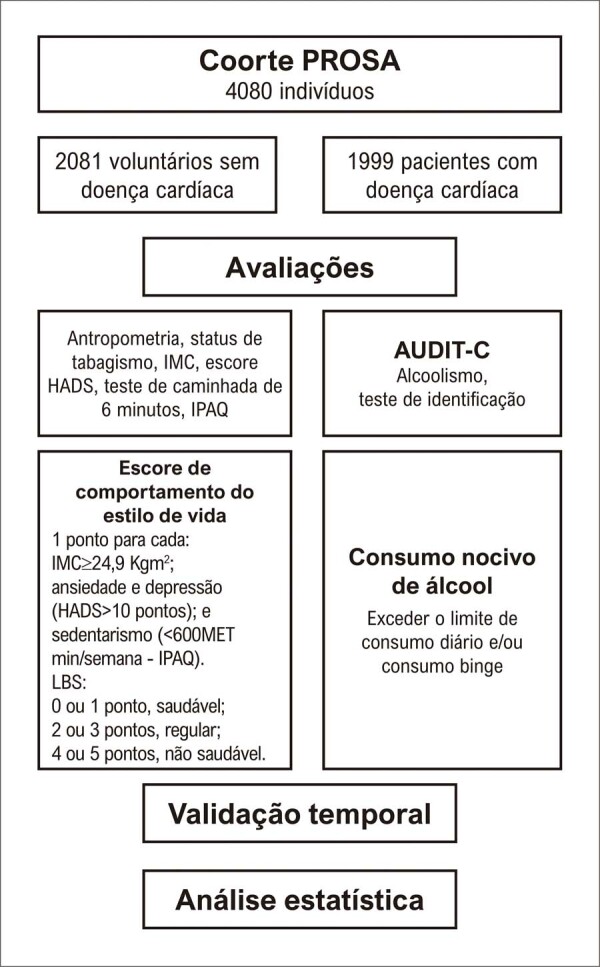
– Fuxograma do estudo; IMC: Índice de Massa Corporal; HADS: Hospital Anxiety and Depression Scale; IPAQ: International Physical Activity Questionnaire; AUDIT-C Alcohol Use Disorders Identification Test; LBS: Escore de estilo de vida; MET: Metabolic Equivalent of Task.

O LBS foi construído atribuindo-se um ponto para cada uma das seguintes variáveis: tabagismo; sobrepeso ou obesidade (Índice de Massa Corporal, IMC ≥ 24,9 Kg/m^2^); ansiedade (pontuação HADS > 10); depressão (pontuação HADS > 10); e sedentarismo (menos de 600 MET-minutos/semana segundo o IPAQ) (Figura 1). O comportamento de estilo de vida saudável foi pontuado como 0 ou 1 ponto, o estilo de vida regular como 2 ou 3 pontos, e o estilo de vida não saudável como 4 ou 5 pontos. Os dados do estudo foram coletados e gerenciados utilizando o REDCap – uma ferramenta de captura eletrônica de dados para pesquisa hospedada no HCFMUSP.^24^

Todos os dados foram coletados no basal e no final do estudo. O estudo foi planejado para um período de três anos de seguimento.

### Desfechos do estudo

O desfecho primário foi a correlação entre os escores do LBS e a incidência de CNA entre pacientes com DC. Foram realizadas análises estatísticas para avaliar a capacidade preditiva do modelo LBS na estimativa das taxas de CNA nas coortes de pacientes com DC e de voluntários. O desfecho secundário envolveu o monitoramento das mudanças no LBS ao longo do tempo e a análise de sua relação com as variações nos níveis de CNA. Isso incluiu o acompanhamento longitudinal para avaliar como as flutuações no LBS correspondem a mudanças no comportamento de consumo de álcool.

### Análise estatística e tamanho da amostra

Dado o número limitado de dados disponíveis na literatura sobre o *Lifestyle Behavior Score* (LBS) e o CNA, não foi realizado cálculo formal do tamanho amostral. Em vez disso, foram definidas, por conveniência, duas grandes coortes de 2000 indivíduos cada.

As variáveis foram expressas como frequências e porcentagens ou médias e desvios padrão, conforme apropriado. A normalidade dos dados foi avaliada pelo teste de Shapiro–Wilk, e as diferenças entre os grupos foram analisadas utilizando o teste t para amostras independentes ou o teste do qui-quadrado. Além disso, a maioria das variáveis foi registrada em formatos dicotômicos ou categóricos, com base em pontos de corte consensuais estabelecidos ou julgamento clínico.

O modelo de treinamento para previsão do CNA com base no LBS foi avaliado por meio de regressão logística multivariada com abordagem *stepwise*. As variáveis foram selecionadas para inclusão nos modelos logísticos com base em dois critérios: valor de p inferior a 0,25 na análise univariada e ausência de colinearidade. A qualidade de ajuste foi avaliada pelo teste de Hosmer-Lemeshow (HL). A validação interna foi realizada utilizando a técnica de *bootstrap* logístico com 1000 réplicas amostrais. As capacidades de discriminação do modelo foram avaliadas por meio de curvas ROC (*Receiver Operating Characteristic*), sensibilidade e especificidade.

**Figura 2:**
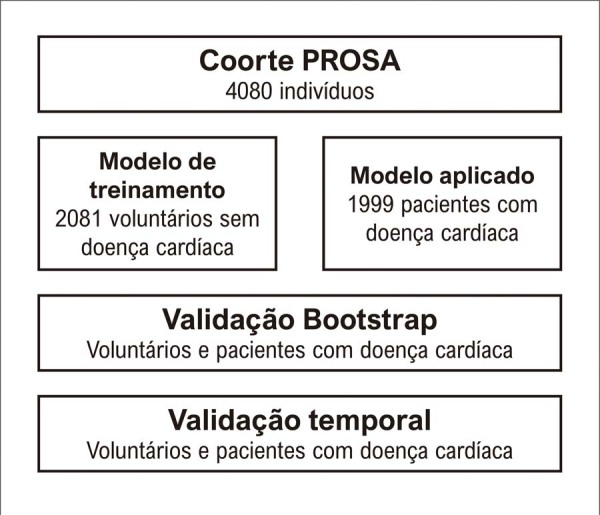
– Fluxograma de validação temporal do modelo do escore de comportamento de estilo de vida para predição do consumo nocivo de álcool.

O modelo preditivo para CNA com base no LBS foi inicialmente desenvolvido com dados de voluntários e, posteriormente, aplicado a pacientes com DC. A validação temporal foi realizada para avaliar a generalização e aplicabilidade do LBS na predição de CNA. Nesse contexto, um subgrupo aleatório da coorte total foi reavaliado três anos após os exames basais (Figura 2). Esse delineamento foi parcialmente influenciado por restrições operacionais durante a pandemia de COVID-19. A ausência de reavaliação em parte da amostra está alinhada com o conceito de “ausência completamente aleatória” (MCAR, do inglês *missing completely at random*).^25^ Para sustentar a hipótese de MCAR, foi confirmado que a falta de acompanhamento não estava associada às características basais ou variáveis de desfecho em nenhuma das fases, conforme avaliado por meio de regressão de modelo misto. Nesse sentido, a validação temporal foi conduzida tanto para os voluntários e pacientes com DC em conjunto, quanto apenas para os pacientes com DC. Testamos a capacidade do modelo logístico multivariado com efeitos aleatórios de considerar medições repetidas durante o acompanhamento, comparando-o ao modelo de regressão logística multivariado que utilizou dados do momento posterior para avaliar a validade temporal do modelo preditivo. Além disso, utilizamos o teste do qui-quadrado para investigar a relação entre alterações no LBS e mudanças associadas no CNA. Um valor de p <0,05 foi considerado estatisticamente significativo.

Os dados foram analisados usando o *software* STATA (Stata Corp LP, College Station, TX, USA) versão 16.

## Resultados

### Características basais

Um total de 4080 indivíduos foi incluído no estudo, sendo 1999 (49,0%) com DC e 2081 (51,0%) voluntários. Entre eles, 341 (17,1%) participantes da coorte com DC e 771 (37,0%) do grupo de voluntários foram classificados como apresentando CNA. A média de idade da população total foi de 57,7 ± 16 anos. Participantes do sexo masculino foram mais prevalentes na coorte com DC (64,3%) em comparação ao grupo de voluntários (40,0%). A coorte com DC era mais velha, apresentava menor nível de escolaridade, mas distribuição de renda semelhante à dos voluntários. Todas as características demográficas e clínicas da população do estudo estão detalhadas na Tabela 1.

**Tabela 1:** – Características basais da coorte do estudo

Variável	População N = 4080	Doença cardíaca n = 1999	Voluntários n = 2081
**Idade, anos ± DP**	57,9±16,0	63,5±14,4	45,6±14,0
**Sexo masculino – n (%)**	2116 (51,9)	1283 (64,3)	818 (40,0)
**Escolaridade – n (%)**			
Fundamental	1411 (34,6)	1018 (50,9)	393 (18,9)
Ensino médio	1446 (35,4)	529 (26,5)	917 (44,1)
Ensino superior	968 (23,7)	340 (17,0)	628 (30,2)
Pós-graduação	164 (3,9)	37 (1,9)	127 (6,1)
Renda familiar mensal^*^ – n (%)			
< $523	377 (9,2)	165 (8,3)	212 (10,2)
$523-1,568	2529 (62,0)	1344 (67,2)	1185 (56,9)
$1,568-$2,613	683 (16,7)	335 (16,8)	348 (16,7)
> $2,613	434 (10,6)	140 (7,0)	294 (14,1)
**Hipertensão – n (%)**	1612 (39,5)	1275 (63,8)	337 (16,2)
**Dislipidemia (n, %)**	941 (23,0)	823 (41,2)	118 (5,7)
**Diabetes – n (%)**	666 (16,3)	563 (28,2)	103 (4,9)
**DRC – n (%)**	91 (2,2)	91 (4,6)	-
**DPOC – n (%)**	27 (0,7)	27 (1,4)	-
**Tabagismo – n (%)**	455 (11,1)	200 (10,0)	255 (12,3)
**Obesidade – n (%)**	1106 (27,1)	472 (23,6)	634 (30,5)
**Ansiedade – n (%)**	321 (7,9)	158 (7,9)	163 (7,8)
**Depressão – n (%)**	132 (3,2)	69 (3,5)	63 (3,0)
**Sedentarismo – n (%)**	1572 (38,5)	854 (42,7)	578 (27,8)
**Escore de estilo de vida – n (%)**			
Saudável	1572 (38,6)	720 (36,0)	852 (40,9)
Regular	1629 (40,0)	821 (41,1)	808 (38,8)
Não saudável	803 (19,7)	415 (20,8)	388 (18,6)

DP: desvio padrão; DPOC: doença pulmonar obstrutiva crônica; DRC: doença renal crônica. *reais convertidos em dólares americanos com base na paridade do poder de compra de 2023 (USD = 2,526).

### Preferências de consumo de bebidas alcoólicas

Padrões de consumo de bebidas alcoólicas categorizados por tipo de bebida para ambas as coortes são apresentados na Tabela 2. A cerveja foi a bebida mais consumida em todos os níveis de ingestão de álcool, seguida pelo vinho e pelos destilados.

**Tabela 2:** – Tipos de bebidas mais consumidas segundo padrão de consumo de álcool (nocivo ou não nocivo) em pacientes com doença cardíaca e voluntários

Tipo de bebida	Pacientes com doença cardíaca			Voluntários		
	Consumo não nocivo de álcool	CNA	p	Consumo não nocivo de álcool	CNA	p
	n = 1598	n = 341		n = 1370	n = 771	
	n (%)	n (%)		n (%)	n (%)	
Cerveja	383 (24,0)	144 (42,2)	<0,001	352 (25,7)	278 (36,1)	0,148
Vinho	220 (13,8)	11 (3,2)	<0,001	249 (18,2)	31 (4,0)	<0,001
Destilado	50 (3,1)	9 (2,6)	0,273	47 (3,4)	18 (2,3)	0,019
Variado	628 (39,3)	175 (51,3)	0,409	422 (30,8)	441 (57,2)	<0,001

CNA: consumo nocivo de álcool.

### Associação entre comportamento de estilo de vida e consumo nocivo de álcool

Entre os voluntários, o UMA foi mais prevalente entre os participantes do sexo masculino. A média de idade dos participantes foi significativamente menor no grupo com CNA que no grupo de voluntários. Os preditores independentes de CNA entre os voluntários estão apresentados na Tabela 3. Entre os pacientes com DC, foram observados resultados semelhantes – a análise multivariada identificou os mesmos preditores independentes associados ao CNA. Esses preditores incluem sexo masculino (razão de chances, OR = 2,95, p < 0,0001), idade (OR = 0,95, p < 0,0001), comportamento de estilo de vida regular (OR = 1,35, p = 0,04) e estilo de vida não saudável (OR = 1,76, p = 0,001) (Tabela 4). A idade mostrou efeito protetor contra o CNA. O modelo apresentou forte poder discriminativo, com estatística GFHL de 0,97 e uma curva ROC de 0,75, atingindo sensibilidade de 60% e especificidade de 75%. Em relação à validade interna, o modelo de regressão por *bootstrap* produziu resultados de sensibilidade consistentes com aqueles obtidos na análise primária da coorte de pacientes com DC.

**Tabela 3:** – Fatores independentes associados com consumo nocivo de álcool em voluntários

Variável (n, %)	Consumo não nocivo de álcool	CNA	Univariada				Multivariada				*Bootstrap*			
				IC95%				IC95%				IC95%		
	n=1309	n=772	OR	Inferior	Superior	p	OR	Inferior	Superior	p	OR	Inferior	Superior	p
**Idade**	48,5±13,7	40,7±12,9				<,0001	0,96	0,95	0,96	<,0001	0,96	0,95	0,96	<,0001
**Sexo masculino**	403 (30,8)	428 (55,4)	2,80	2,32	3,36	<,0001	3,00	2,46	3,68	<,0001	3,00	2,46	3,68	<,0001
**Escolaridade**														
Fundamental	470 (35,9)	285 (36,9)				,02								
Ensino médio	557 (42,6)	359 (46,5)	1,81	0,50	6,64	,36								
Ensino superior	269 (20,6)	122 (15,8)	2,58	0,72	9,20	,15								
Pós-graduação	12 (0,9)	3 (0,3)	2,42	0,88	8,67	,17								
**Renda familiar**														
< 1 salário	117 (8,9)	95 (12,7)				,02								
1 - 3 salários	765 (58,4)	418 (54,1)	0,67	0,50	0,90	,009								
3 - 5 salários	221 (16,9)	126 (16,3)	0,70	0,50	1,00	,05								
> 5 salários	279 (9,4)	155 (14,2)	0,87	0,61	1,25	,45								
**Hipertensão**	226 (17,3)	109 (14,1)	0,78	0,61	1,01	,06								
**Dislipidemia**	88 (6,7)	27 (3,5)	0,51	0,32	0,78	,002								
**Diabetes**	72 (5,5)	29 (2,8)	0,66	0,42	1,02	,06								
**Estilo de vida**														
Saudável	176 (13,5)	69 (8,9)				,003								
Regular	323 (24,7)	202 (26,1)	1,29	1,05	1,57	0,01	1,32	1,06	1,65	,01	1,32	1,07	1,63	,009
Não saudável	23 (1,76)	16 (2,1)	1,39	1,09	1,78	0,009	1,47	1,12	1,93	,005	1,48	1,12	1,95	,006

IC: intervalo de confiança; OR: odds ratio; CNA: consumo nocivo de álcool; valores p <0,05 foram considerados estatisticamente significativos.

**Tabela 4:** – Fatores independentes associados com consumo nocivo de álcool em pacientes com doença cardíaca

Variável (n, %)	Consumo não nocivo de álcool	CNA	Univariada				Multivariada				*Bootstrap*			
				IC95%				IC95%				IC95%		
	n=1659	n=340	OR	Inferior	Superior	p	OR	Inferior	Superior	p	OR	Inferior	Superior	p
**Idade**	65,3±13,6	54,5±14,8				< 0,0001	0,95	0,94	0,96	<0,0001	0,95	0,94	0,96	<0,0001
**Sexo masculino**	1008 (65,6)	277 (81,5)	2,84	2,12	3,80	< 0,0001	2,95	2,17	3,99	<0,0001	2,95	2,16	4,03	<0,0001
**Escolaridade**														
Fundamental	873 (52,6)	187 (55)				< 0,0001								
Ensino médio	368 (22,2)	162 (47,6)	2,10	1,61	2,62	< 0,0001								
Ensino superior	254 (15,3)	86 (25,3)	1,18	2,11	9,20	0,002								
Pós-graduação	27 (1,6)	10 (2,9)	1,72	0,82	3,63	0,15								
**Renda familiar**														
< 1 salário	137 (8,3)	28 (8,2)				0,06								
1 - 3 salários	1133(68,3)	213 (62,6)	0,92	0,60	1,41	0,70								
3 - 5 salários	231 (13,9)	58 (17,0)	1,22	0,74	2,02	0,42								
> 5 salários	107 (6,5)	33 (9,7)	1,50	0,86	2,65	0,15								
**Hipertensão**	1104 (66,5)	173 (51,0)	0,52	0,41	0,66	< 0,0001								
**Dislipidemia**	706 (42,5)	120 (35,3)	0,73	0,58	0,94	0,01								
**Diabetes**	494 (30,0)	70 (20,6)	0,61	0,47	0,81	0,001								
**Estilo de vida**														
Saudável	616 (37,1)	105 (31,0)				0,009								
Regular	711 (42,9)	144 (42,4)	1,19	0,90	1,56	0,21	1,34	1,01	1,80	0,04	1,35	1,01	1,80	0,040
Não saudável	325 (19,6)	90 (26,5)	1,63	1,19	2,22	0,002	1,75	1,26	2,44	0,001	1,76	1,25	1,46	0,001

IC: intervalo de confiança; OR: odds ratio; CNA: consumo nocivo de álcool; valores p <0,05 foram considerados estatisticamente significativos.

### Modelos de validação temporal

**Figura 3:**
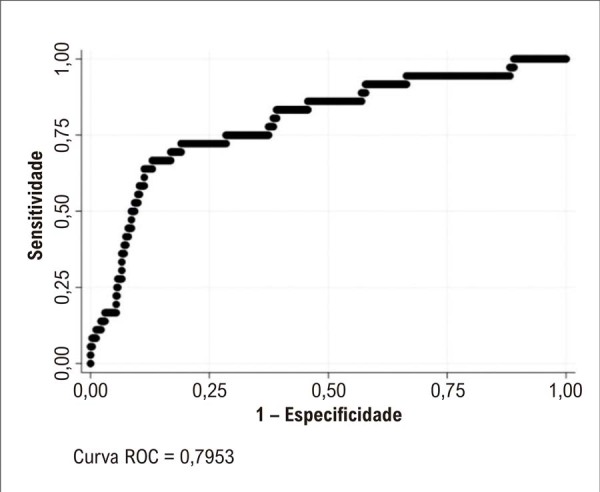
– Curva ROC (Receiver Operating Characteristic) dos pacientes com doença cardíaca associada ao consumo nocivo de álcool.

Na avaliação de validação temporal, o modelo logístico multivariado apresentou um forte desempenho discriminativo, com uma curva ROC de 0,78, sensibilidade de 57% e especificidade de 77%. No subgrupo de pacientes com DC, a discriminação do modelo foi ainda mais acentuada, alcançando uma ROC de 0,80, sensibilidade de 72% e especificidade de 81% (Figura 3). O modelo multivariado de efeitos aleatórios não melhorou a capacidade preditiva do modelo de validação temporal (teste de razão de verossimilhança: p=0,07 com uma ROC de 0,76) quando aplicado à coorte completa, confirmando a estabilidade do modelo ao longo do tempo, independentemente das características basais dos pacientes reavaliados, e consistente com o padrão MCAR (de perda de seguimento.

### Alterações nos escores de comportamento de estilo de vida estão associadas a variações no consumo nocivo de álcool

Além disso, entre os pacientes que passaram por duas avaliações, uma melhora no LBS para um status mais saudável (observada em 13,2% dos pacientes) foi seguida por uma redução no CNA (7,9%, p=0,02) após um período de acompanhamento de três anos. A mesma tendência foi observada em pacientes cujo LBS piorou, com um aumento no CNA (3,8% vs. 7,0%, p=0,01).

A Figura Central ilustra as principais conclusões do estudo.

## Discussão

Este manuscrito apresenta um novo modelo de LBS, demonstrando uma robusta capacidade preditiva para identificar o CNA na população geral, especialmente em indivíduos com DC. O desenvolvimento dessa ferramenta enfatiza a importância de categorizar os comportamentos relacionados ao estilo de vida, particularmente no contexto do manejo do risco para DC. As descobertas do nosso estudo vão ao encontro de pesquisas existentes na população geral, revelando uma correlação consistente entre comportamentos não saudáveis – como tabagismo, sedentarismo e saúde mental precária—e maiores taxas de abuso de álcool.^14-18,26-28^

Em nosso estudo, observamos que indivíduos mais velhos, especialmente aqueles com DC, apresentaram uma menor tendência ao CNA. O efeito protetor do envelhecimento contra o HAC em pacientes com DC é mais pronunciado do que o observado em voluntários (5% vs. 4%, respectivamente). Esse achado sugere uma maior conscientização da população com HD sobre a importância de reduzir os fatores de risco.

A proporção de homens com CNA tende a ser maior do que a de mulheres na população geral. Embora essa tendência tenha diminuído ao longo dos anos, nosso estudo revelou que o risco de HAC continua elevado entre os homens, tanto em voluntários quanto em pacientes com DC.^29^

Em nosso escore de LBS, consideramos o IMC como um indicador dos padrões alimentares. Curiosamente, o Departamento de Agricultura dos Estados Unidos (USDA), embora não inclua bebidas alcoólicas como um componente do padrão alimentar, enfatiza a importância de contabilizar as calorias presentes nas bebidas para manter limites alimentares saudáveis. O não cumprimento desses limites pode contribuir para um estilo de vida desfavorável.^30^

Além disso, consideramos a atividade física como um fator de estilo de vida com impacto significativo no consumo de álcool. Um estudo recente relatou um aumento significativo nos casos de consumo não nocivo de álcool entre aqueles com níveis mais elevados de aptidão física. Em nossa opinião, esses achados estão alinhados com o conceito de que o consumo não nocivo de álcool faz parte de um estilo de vida saudável.^31,32^ Além disso, o uso de tabaco, o qual está fortemente associado ao CNA,^15-18^ também foi incluído no escore de LBS.

Nosso estudo também incorporou o comportamento psicossocial como um componente do escore, reconhecendo sua interação significativa com o consumo de álcool e seu impacto no estilo de vida. A relação entre o consumo de álcool e o estresse é complexa. Embora o álcool possa aliviar temporariamente a ansiedade, ele também pode atuar como um fator estressor, aumentando a probabilidade de CNA em resposta ao estresse, o que é frequentemente denominado como consumo excessivo de álcool relacionado ao estresse.^27^ Além disso, a depressão tem sido associada ao CNA, particularmente entre estudantes universitários.^28^

Em resumo, a heterogeneidade dos fatores relevantes mencionados acima justifica o desenvolvimento de um sistema de pontuação como o que aplicamos na população geral e em pacientes com DC.^9^ Na prática, para essa população pouco estudada, os resultados do nosso escore podem orientar intervenções voltadas à melhoria do estilo de vida para combater o CNA, como programas de cessação do tabagismo, promoção da atividade física e terapias para saúde mental.

Uma limitação deste estudo é a não inclusão da avaliação do comportamento do sono em nosso escore. Embora o comportamento do sono tenha sido relatado como um fator de estilo de vida influente, sua associação com o CNA ainda não apresenta um impacto significativo quando comparado aos outros fatores incluídos no nosso escore de LBS. Outra limitação deste estudo é a ausência de validação externa formal do modelo. No entanto, esse potencial viés pode ter sido minimizado pela validação temporal realizada, que reduz a probabilidade de viés em grandes populações, especialmente em grupos homogêneos, como os pacientes com DCs.^33^

## Conclusões

Este estudo mostrou que o LBS, um novo escore e estilo de vida, é um preditor confiável de CNA em pacientes com DC. Ainda, observamos que uma melhora no comportamento de estilo de vida pode ajudar a reduzir o CNA.

## Material suplementar:

Supplemental Material Para informação adicional, por favor, clique aqui.
