# Supplementary material for: Consumo Abusivo de Álcool em Cardiopatas: Análise de Risco pelo Comportamento de Estilo de Vida – Projeto PROSA (Projeto Saúde e Álcool)
Source: Arq Bras Cardiol. 2025 Oct 9;122(10):e20240744. [Article in Portuguese] doi: 10.36660/abc.20240744 (PMC12677455; doi:10.36660/abc.20240744)
Supplement: Supplemental Material [file 0066-782x-abc-122-10-e20240744-suppl01.pdf]

Consumo abusivo de álcool em cardiopatas: análise de risco pelo comportamento de estilo de vida – Projeto PROSA (PROjeto Saúde e Álcool)

Harmful alcohol consumption in heart disease patients: a risk analysis by lifestyle behavior - The PROSA Project (PROjeto **S**aúde e **Á**lcool**)**

Supplemental Material

Contents

AUDIT-C 2

Institutional Q-PROSA questionnaire: 3

AUDIT-C

Here is an English translation of the AUDIT-C used in Brazil and built using REDCap electronic data capture tools hosted at Hospital das Clínicas da Faculdade de Medicina da Universidade de São Paulo (HC-FMUSP). The Portuguese version of the questionnaire is available at:

https://edisciplinas.usp.br/pluginfile.php/4170599/mod_resource/content/1/audit.pdf

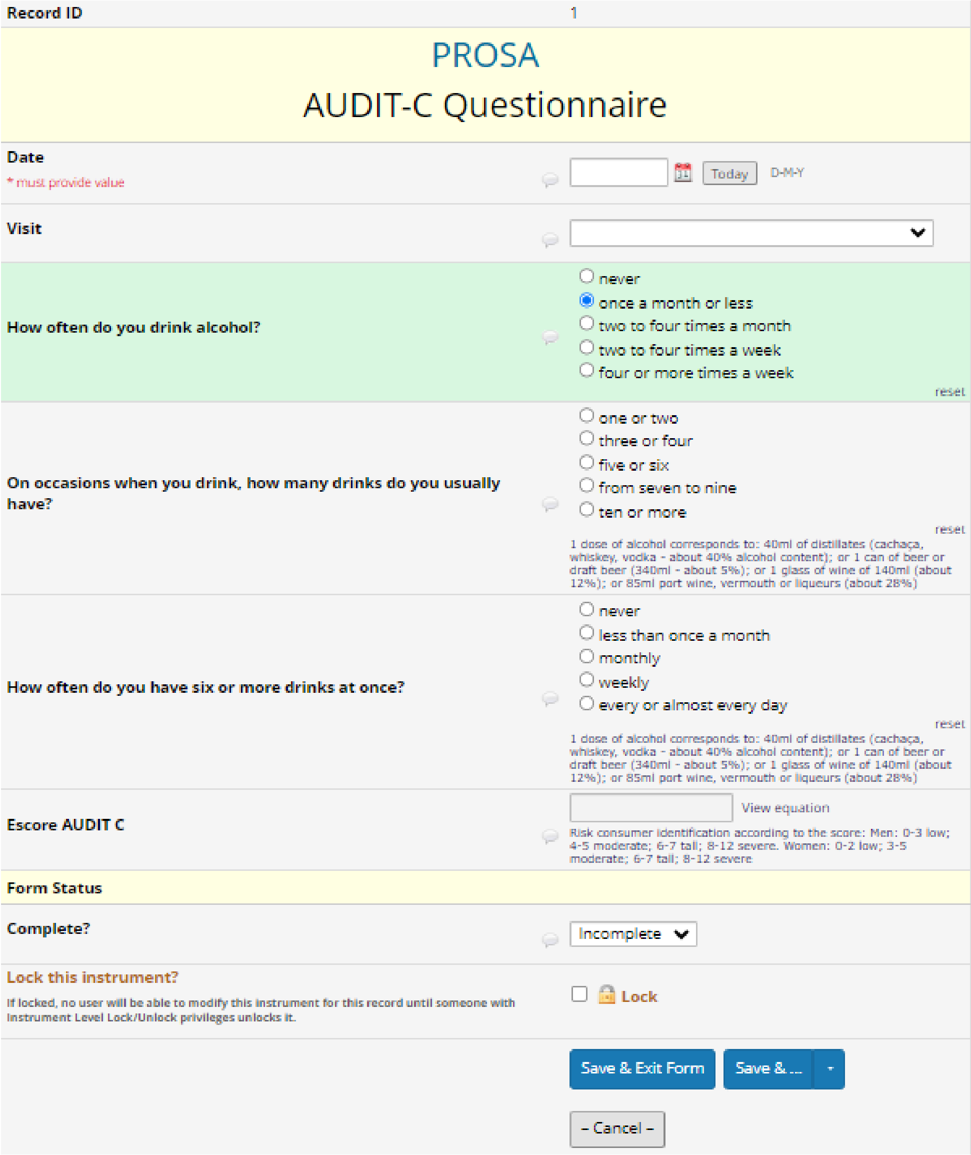

Record ID 1

PROSA

AUDIT-C Questionnaire

Date
* must provide value
Today D-M-Y

Visit

How often do you drink alcohol?
- never
- once a month or less
- two to four times a month
- two to four times a week
- four or more times a week

reset

On occasions when you drink, how many drinks do you usually have?
- one or two
- three or four
- five or six
- from seven to nine
- ten or more

reset

1 dose of alcohol corresponds to: 40ml of distillates (cachaça, whiskey, vodka - about 40% alcohol content); or 1 can of beer or draft beer (340ml - about 5%); or 1 glass of wine of 140ml (about 12%); or 85ml port wine, vermouth or liqueurs (about 28%)

How often do you have six or more drinks at once?
- never
- less than once a month
- monthly
- weekly
- every or almost every day

reset

1 dose of alcohol corresponds to: 40ml of distillates (cachaça, whiskey, vodka - about 40% alcohol content); or 1 can of beer or draft beer (340ml - about 5%); or 1 glass of wine of 140ml (about 12%); or 85ml port wine, vermouth or liqueurs (about 28%)

Escore AUDIT C
View equation
Risk consumer identification according to the score: Men: 0-3 low; 4-5 moderate; 6-7 tall; 8-12 severe. Women: 0-2 low; 3-5 moderate; 6-7 tall; 8-12 severe

Form Status

Complete? Incomplete

Lock this instrument?
If locked, no user will be able to modify this instrument for this record until someone with Instrument Level Lock/Unlock privileges unlocks it.
Lock

Save & Exit Form | Save & ...

- Cancel -

Institutional Q-PROSA questionnaire:

Assess the amount (weekly frequency and typical dose per day of consumption) by type of drink (beer, wine, and spirits). Built using REDCap electronic data capture tools hosted at Hospital das Clínicas da Faculdade de Medicina da Universidade de São Paulo (HC-FMUSP).

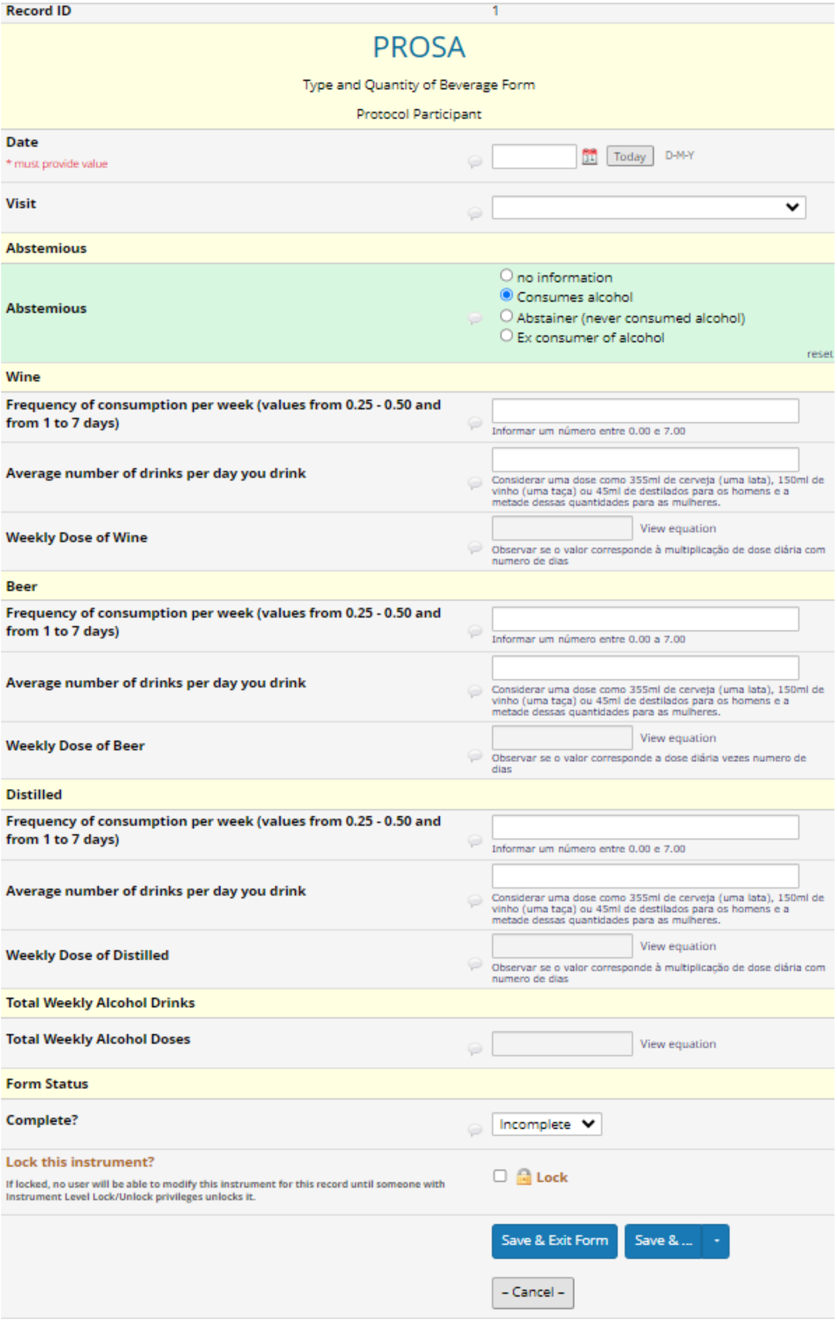

Record ID	1
**PROSA**<br>Type and Quantity of Beverage Form<br>Protocol Participant	
Date<br>* must provide value	Today D-M-Y
Visit	
**Abstemious**	
Abstemious	no information<br>Consumes alcohol<br>Abstainer (never consumed alcohol)<br>Ex consumer of alcohol<br>reset
**Wine**	
Frequency of consumption per week (values from 0.25 - 0.50 and from 1 to 7 days)	Informar um número entre 0.00 e 7.00
Average number of drinks per day you drink	Considerar uma dose como 355ml de cerveja (uma lata), 150ml de vinho (uma taça) ou 45ml de destilados para os homens e a metade dessas quantidades para as mulheres.
Weekly Dose of Wine	View equation<br>Observar se o valor corresponde à multiplicação de dose diária com numero de dias
**Beer**	
Frequency of consumption per week (values from 0.25 - 0.50 and from 1 to 7 days)	Informar um número entre 0.00 a 7.00
Average number of drinks per day you drink	Considerar uma dose como 355ml de cerveja (uma lata), 150ml de vinho (uma taça) ou 45ml de destilados para os homens e a metade dessas quantidades para as mulheres.
Weekly Dose of Beer	View equation<br>Observar se o valor corresponde a dose diária vezes numero de dias
**Distilled**	
Frequency of consumption per week (values from 0.25 - 0.50 and from 1 to 7 days)	Informar um número entre 0.00 e 7.00
Average number of drinks per day you drink	Considerar uma dose como 355ml de cerveja (uma lata), 150ml de vinho (uma taça) ou 45ml de destilados para os homens e a metade dessas quantidades para as mulheres.
Weekly Dose of Distilled	View equation<br>Observar se o valor corresponde à multiplicação de dose diária com numero de dias
**Total Weekly Alcohol Drinks**	
Total Weekly Alcohol Doses	View equation
**Form Status**	
Complete?	Incomplete
Lock this instrument?<br>If locked, no user will be able to modify this instrument for this record until someone with Instrument Level Lock/Unlock privileges unlocks it.	Lock
	Save & Exit Form Save & ...<br>– Cancel –
