# Supplementary material for: Consumo Abusivo de Álcool em Cardiopatas: Análise de Risco pelo Comportamento de Estilo de Vida – Projeto PROSA (Projeto Saúde e Álcool)
Source: Arq Bras Cardiol. 2025 Oct 9;122(10):e20240744. [Article in Portuguese] doi: 10.36660/abc.20240744 (PMC12677455; doi:10.36660/abc.20240744)
Supplement: Translation

# Translation: Harmful Alcohol Consumption in Patients with Heart Disease: A Risk Analysis by Lifestyle Behavior – The Brazilian PROSA Project (Projeto Saúde e Álcool)

**DOI:** 10.36660/abc.20240744i

**Authors:** Martino Martinelli-Filho, Caio Vitale Spaggiari, Roberta Vanalli Baroni, Cinthya Ibrahim Guirao Gomes, Thiago Ovanessian Hueb, Sergio Augusto Mezzalira Martins, Anísio Alexandre Andrade Pedrosa, Silvana Angelina Dório Nishioka, Sergio Freitas de Siqueira

**Affiliations:** 1 Brazil Instituto do Coração do Hospital das Clínicas da Faculdade de Medicina da Universidade de São Paulo, São Paulo, SP – Brazil

**Keywords:** Alcohol Consumption, Life Style, Heart Diseases

## Abstract

**Background:**

Moderate alcohol intake appears to have a protective cardiovascular effect, whereas harmful alcohol consumption (HAC) is associated with a high rate of chronic diseases and mortality. The amount of alcohol intake has been associated with lifestyle behaviors; however, this relationship has not been assessed in patients with heart disease (HD).

**Objective:**

To evaluate the association between lifestyle behavior and HAC in HD patients.

**Methods:**

This is an observational study that included one group of patients with HD and one group of volunteers designed to assess the association of HAC with lifestyle behavior using a score of lifestyle behavior (the LBS score). We assessed alcohol consumption using the AUDIT-C test. LBS was developed by assigning 1 point to each variable – smoking; overweight or obese (body mass index ≥24.9); anxiety; depression and lack of physical activity, and classified into healthy (0/1 point), regular (2/3 points), or unhealthy (4/5 points). Statistical analysis adopted as a significance level p-value <0.05.

**Results:**

The study included 1,999 patients with HD and 2,081 volunteers. Regression analysis controlled for age and sex, revealed that regular and unhealthy LBS are independent predictors of HAC (OR= 1.35, p=0.04 and OR= 1.76, p=0.001, respectively) in HD patients, similar as volunteers. Good discrimination capability of the regression model was reported in the temporal validation (ROC 0.80). Furthermore, LBS improvements were associated with HAC reductions (p=0.02).

**Conclusions:**

This study showed that risk assessment for HAC using a new lifestyle score is a reliable predictor in HD patients. Also, it was observed that improving lifestyle behavior may help to reduce HAC.

## Introduction

Numerous studies have suggested potential cardiovascular benefits of light-to-moderate alcohol consumption in general population, indicating a protective effect against cardiovascular diseases.^1-4^ However, understanding the true nature of the relationship between alcohol consumption and health outcomes requires consideration of confounding variables, which has not been assessed in patients with heart disease (HD).^5-7^

In this regard, adopting a healthy lifestyle has consistently been shown to reduce the risk of major chronic illnesses, including conditions, including cancer, heart attack, hypertension, stroke, diabetes, and heart rhythm disorders.^1,2^ In contrast, harmful alcohol consumption (HAC) is a known contributor to adverse health outcomes, as it exacerbates chronic diseases and increases both morbidity and mortality.^8-10^ Notably, in patients with HD, the correlation between alcohol consumption and health outcomes follows a dose-response pattern.^11^ In this context, HAC can have severe consequences by aggravating preexisting conditions and posing significant threats to patients’ health.^10-12^

Global health initiatives – exemplified by the World Health Organization (WHO) – have sought to promote research aimed at understanding and reducing the prevalence of HAC across diverse populations.^13^ The key challenge remains in establishing a causal relationship between alcohol consumption and the onset or progression of chronic diseases.

In this context, efforts have been directed toward assessing the potential risks of alcohol consumption, particularly in individuals with pre-existing health conditions, and aligning these risks with lifestyle behaviors. Smoking, for example, not only exhibits a strong association with chronic obstructive pulmonary disease and lung cancer but is also recognized for its correlation with HAC.^14-18^

The development of a population-specific lifestyle assessment tool, the Lifestyle Behavior Score (LBS), is crucial for identifying factors linked to patterns of alcohol consumption and enabling robust statistical analyses tailored to specific populations. Hence, we hypothesize a strong association between an unhealthy LBS and UNA, particularly in patients with HD. Therefore, this study aims to assess the association between LBS and UNA in individuals with and without HD.

## Methods

This is a prospective study that included two distinct cohorts, HD patients and volunteers, categorized by pattern of alcohol consumption.

### Study population

The cohort of patients with HD included clinically stable outpatients diagnosed with any form of cardiomyopathy, who were under the care of the Heart Institute (*Instituto do Coração, InCor*) – General Hospital of the University of São Paulo Medical School (HCFMUSP). The cohort of volunteers was recruited from family members or caregivers of the HD patients, and from hospital staff. Both cohorts included individuals of any sex, gender or race, over 18 years of age. Informed consent was obtained from all study participants, and the study protocol was approved by the Ethics Committee of InCor and of HCFMUSP (CAAE 61741916.0.0000.0068 approval number 1.846.682 on December 2, 2016).

### Alcohol Intake, pattern of alcohol consumption and lifestyle behavior score

To evaluate alcohol intake, we applied the Alcohol Use Disorders Identification Test (AUDIT-C) (Supplemental Material).^19-21^ However, the AUDIT-C does not specify the type of alcoholic beverage consumed, and we then used the institutional questionnaire Q-PROSA (Supplemental Material) to more accurately assess the preferred type of beverage – beer, spirits, and/or wine – and the amounts consumed. Participants were characterized according to the amount of alcohol consumed. HAC was classified as >28g of ethanol per day for men, and >14g of ethanol per day for women or as a binge consumption according to AUDIT-C score.

To assess lifestyle behavior, several factors were assessed using standardized questionnaires. Depression and anxiety levels were assessed using the Hospital Anxiety and Depression Scale (HADS),^22^ and physical activity levels were measured using the International Physical Activity Questionnaire (IPAQ).^23^ Smoking status, schooling, and income were assessed using objective questions. Additionally, measurements of weight and height were obtained in all participants. All the questionnaires used were validated versions for the Brazilian Portuguese language.

The LBS was built by attributing one point for each variable: smoking; overweight or obese (body mass index, BMI ≥ 24.9 Kg/m^2^); anxiety (HADS score > 10 points); depression (HADS score > 10 points); and sedentary (less than 600 MET minutes/week per IPAQ) (Figure 1). Healthy lifestyle behavior was scored as 0 or 1 point, regular lifestyle was scored as 2 or 3 points, and unhealthy lifestyle was scored as 4 or 5 points. Study data were collected and managed using REDCap – a Research Electronic Data Capture tool hosted at HCFMUSP.^24^

**Figure 1:** – Study flowchart. BMI: body mass index; HADS: Hospital Anxiety and Depression Scale; IPAQ: International Physical Activity Questionnaire; LBS: Lifestyle Behavior Score; MET: Metabolic Equivalent of Task.

All data were collected at baseline and at the end of the study. The study was planned for a three-year follow-up period.

### Study endpoints

The primary endpoint was the correlation between the LBS scores and the incidence of HAC among patients with HD. Statistical analyses were performed to assess the predictive capacity of the LBS model in estimating HAC rates across both the HD and volunteer cohorts. The secondary endpoint involved monitoring changes in LBS over time and analyzing their relationship with variations in HAC levels. This included longitudinal tracking to evaluate how fluctuations in LBS correspond to shifts in alcohol consumption behavior.

### Statistical analysis and sample size

Given the limited data available in the literature on LBS and HAC, no formal sample size calculation was conducted. Instead, two large cohorts of 2,000 individuals each were defined for convenience.

Variables were expressed as frequencies and percentages or means and standard deviations, as appropriate. Normality of the data was assessed using the Shapiro–Wilk test, and group differences were evaluated using the independent-sample t-test or chi-square test. Additionally, most variables were recorded into dichotomous or categorical formats based on established consensus cutoffs or clinical judgment.

The training model for predicting HAC by LBS was assessed using the multivariate logistic regression model with a stepwise approach. Variables were selected for inclusion in the logistic models based on two criteria: a p value of less than 0.25 in the univariate analysis and the absence of collinearity. The goodness of fit was evaluated using the Hosmer-Lemeshow test (GFHL). Internal validation was performed using a logistic bootstrap technique with 1000 sample replications. The model’s discrimination capabilities were assessed using receiver operating characteristic (ROC) curves, sensitivity, and specificity.

The predictive model for HAC using LBS was initially developed with data from volunteers and subsequently applied to HD patients. Temporal validation was conducted to assess the generalizability and applicability of LBS for predicting HAC. In this context, a random subgroup of the overall cohort underwent evaluation three years after their baseline assessments (Figure 2). This design was partly shaped by operational constraints during the COVID-19 pandemic. The absence of reevaluation in part of the sample aligns with the concept of ‘missing completely at random’ (MCAR).^25^ To support the MCAR assumption, we confirmed that the lack of follow-up was not associated with baseline characteristics or outcome variables in either phase, as assessed by a mixed-model regression. In this regard, temporal validation was conducted for both volunteers and HD patients together, as well as for HD patients alone. We tested the logistic multivariate random-effects model’s ability to account for repeated measurements during follow-up evaluation, comparing it to the multivariate logistic regression model, which utilized data from the later time point to assess the temporal validity of the predictive model. Furthermore, we used the chi-square test to investigate the relationship between alterations in LBS and associated changes in HAC. P value <0.05 was considered statistically significant.

**Figure 2:** – Temporal validation flowchart of the Lifestyle Behavior Score Model for predicting Harmful Alcohol Consumption.

All data were analyzed using STATA (Stata Corp LP, College Station, TX, USA) software version 16.

## Results

### Baseline characteristics

A total of 4,080 individuals were included in the study, 1,999 (49.0%) with HD and 2,081 (51.0%) volunteers. Among them, 341 (17.1%) subjects in the HD cohort and 771 (37.0%) in the volunteer group were classified as exhibiting HAC. The mean age of the total population was 57.7 ± 16 years. Male participants were more prevalent in the HD cohort (64.3%) compared to the volunteer group (40.0%). The HD cohort was older, had lower educational attainment, but a similar income distribution when compared to volunteers. All demographic and clinical characteristics of the study population are detailed in Table 1.

**Table 1:** – Baseline characteristics of the study cohort

Variable	Population N = 4080	Heart Disease n = 1999	Volunteers n = 2081
**Age – years±SD**	57.9±16.0	63.5±14.4	45.6±14.0
**Male Sex – n (%)**	2116 (51.9)	1283 (64.3)	818 (40.0)
**Schooling – n (%)**			
Elementary	1411 (34.6)	1018 (50.9)	393 (18.9)
High School	1446 (35.4)	529 (26.5)	917 (44.1)
University education	968 (23.7)	340 (17.0)	628 (30.2)
Postgraduate	164 (3.9)	37 (1.9)	127 (6.1)
Monthly Household Income* – n (%)			
Less than $523	377 (9.2)	165 (8.3)	212 (10.2)
$523-1,568	2529 (62.0)	1344 (67.2)	1185 (56.9)
$1,568-$2,613	683 (16.7)	335 (16.8)	348 (16.7)
More than $2,613	434 (10.6)	140 (7.0)	294 (14.1)
**Hypertension – n (%)**	1612 (39.5)	1275 (63.8)	337 (16.2)
**Dyslipidemia (n, %)**	941 (23.0)	823 (41.2)	118 (5.7)
**Diabetes – n (%)**	666 (16.3)	563 (28.2)	103 (4.9)
**CKD – n (%)**	91 (2.2)	91 (4.6)	-
**COPD – n (%)**	27 (0.7)	27 (1.4)	-
**Smoking – n (%)**	455 (11.1)	200 (10.0)	255 (12.3)
**Obesity – n (%)**	1106 (27.1)	472 (23.6)	634 (30.5)
**Anxiety – n (%)**	321 (7.9)	158 (7.9)	163 (7.8)
**Depression – n (%)**	132 (3.2)	69 (3.5)	63 (3.0)
**Sedentary – n (%)**	1572 (38.5)	854 (42.7)	578 (27.8)
**Lifestyle Score – n (%)**			
Healthy	1572 (38.6)	720 (36.0)	852 (40.9)
Regular	1629 (40.0)	821 (41.1)	808 (38.8)
Unhealthy	803 (19.7)	415 (20.8)	388 (18.6)

SD: standard deviation, COPD: chronic obstructive pulmonary disease, CKD: chronic kidney disease. *Brazilian Reais (R$) were converted to U.S. Dollar (USD) based on the purchasing power parity (PPP 2023: USD = 2.526).

### Alcoholic beverage preferences

Drinking patterns categorized by beverage type for both cohorts are presented in Table 2. Beer was the most consumed beverage across all levels of alcohol intake, followed by wine and spirits.

**Table 2:** – Most commonly consumed types of beverages according to alcohol consumption pattern (harmful or non-harmful) in patients with heart disease and volunteers

	Heart Disease Patients			Volunteers		
	Non-HAC	HAC	p	Non-HAC	HAC	p
	n = 1598	n = 341		n = 1370	n = 771	
Type of beverage	n (%)	n (%)		n (%)	n (%)	
Beer	383 (24.0)	144 (42.2)	<0.001	352 (25.7)	278 (36.1)	0.148
Wine	220 (13.8)	11 (3.2)	<0.001	249 (18.2)	31 (4.0)	<0.001
Spirit	50 (3.1)	9 (2.6)	0.273	47 (3.4)	18 (2.3)	0.019
Variado	628 (39.3)	175 (51.3)	0.409	422 (30.8)	441 (57.2)	<0.001

HAC: Harmful Alcohol Consumption. Mixed means any combination of beverages.

### Association between lifestyle behavior and harmful alcohol consumption

Among volunteers, HAC more prevalent in male participants. Mean age of participants was significantly in the HAC than in the volunteer group. The independent predictors of HAC in volunteers are presented in Table 3. In HD patients, similar findings were observed –multivariate analysis identified the same independent predictors associated with HAC. These included male sex (odds ratio, OR=2.95, P<0.0001), age (OR=0.95, p<0.0001), regular lifestyle behavior (OR=1.35, p=0.04), and unhealthy lifestyle (OR=1.76, p=0.001) (Table 4). Notably, age showed a protective effect against HAC. The model displayed strong discriminative power, with a GFHL statistic of 0.97 and a ROC of 0.75, yielding a sensitivity of 60% and specificity of 75%. Regarding internal validity, the bootstrap regression outcome model produced sensitivity results consistent with those from the primary HD cohort analysis.

**Table 3:** – Independent factors associated with harmful alcohol consumption in heart disease patients

Variable (n, %)	Non-HAC	HAC	Univariate				Multivariate				Bootstrap			
				95.0% CI				95.0% CI				95.0% CI		
	n=1309	n=772	OR	Lower	Upper	p	OR	Lower	Upper	p	OR	Lower	Upper	p
**Age**	48.5±13.7	40.7±12.9				<.0001	0.96	0.95	0.96	<.0001	0.96	0.95	0.96	<.0001
**Male Sex**	403 (30.8)	428 (55.4)	2.80	2.32	3.36	<.0001	3.00	2.46	3.68	<.0001	3.00	2.46	3.68	<.0001
**Schooling**														
Elementary	470 (35.9)	285 (36.9)				.02								
High School	557 (42.6)	359 (46.5)	1.81	0.50	6.64	.36								
University education	269 (20.6)	122 (15.8)	2.58	0.72	9.20	.15								
Postgraduate	12 (0.9)	3 (0.3)	2.42	0.88	8.67	.17								
**Family income**														
Less than 1 income	117 (8.9)	95 (12.7)				.02								
1 to 3 incomes	765 (58.4)	418 (54.1)	0.67	0.50	0.90	.009								
3 to 5 incomes	221 (16.9)	126 (16.3)	0.70	0.50	1.00	.05								
More than 5 incomes	279 (9.4)	155 (14.2)	0.87	0.61	1.25	.45								
**Hypertension**	226 (17.3)	109 (14.1)	0.78	0.61	1.01	.06								
**Dyslipidemia**	88 (6.7)	27 (3.5)	0.51	0.32	0.78	.002								
**Diabetes**	72 (5.5)	29 (2.8)	0.66	0.42	1.02	.06								
**Lifestyle**														
Healthy	176 (13.5)	69 (8.9)				.003								
Regular	323 (24.7)	202 (26.1)	1.29	1.05	1.57	0.01	1.32	1.06	1.65	.01	1.32	1.07	1.63	.009
Unhealthy	23 (1.76)	16 (2.1)	1.39	1.09	1.78	0.009	1.47	1.12	1.93	.005	1.48	1.12	1.95	.006

CI: confidence interval; OR: odds ratio; HAC: Harmful Alcohol Consumption. P value of <.05 were considered statistically significant.

**Table 4:** – Independent factors associated with harmful alcohol consumption in heart disease patients

Variable (n, %)	Non-HAC	HAC	Univariate				Multivariate				Bootstrap			
				95.0% CI				95.0% CI				95.0% CI		
	n=1659	n=340	OR	Lower	Upper	p	OR	Lower	Upper	p	OR	Lower	Upper	p
**Age**	65.3±13.6	54.5±14.8				< 0.0001	0.95	0.94	0.96	<0.0001	0.95	0.94	0.96	<0.0001
**Male Sex**	1008 (65.6)	277 (81.5)	2.84	2.12	3.80	< 0.0001	2.95	2.17	3.99	<0.0001	2.95	2.16	4.03	<0.0001
**Schooling**														
Elementary	873 (52.6)	187 (55)				< 0.0001								
High School	368 (22.2)	162 (47.6)	2.10	1.61	2.62	< 0.0001								
University education	254 (15.3)	86 (25.3)	1.18	2.11	9.20	0.002								
Postgraduate	27 (1.6)	10 (2.9)	1.72	0.82	3.63	0.15								
**Family income**														
Less than 1 income	137 (8.3)	28 (8.2)				0.06								
1 to 3 incomes	1133(68.3)	213 (62.6)	0.92	0.60	1.41	0.70								
3 to 5 incomes	231 (13.9)	58 (17.0)	1.22	0.74	2.02	0.42								
More than 5 incomes	107 (6.5)	33 (9.7)	1.50	0.86	2.65	0.15								
**Hypertension**	1104 (66.5)	173 (51.0)	0.52	0.41	0.66	< 0.0001								
**Dyslipidemia**	706 (42.5)	120 (35.3)	0.73	0.58	0.94	0.01								
**Diabetes**	494 (30.0)	70 (20.6)	0.61	0.47	0.81	0.001								
**Lifestyle**														
Healthy	616 (37.1)	105 (31.0)				0.009								
Regular	711 (42.9)	144 (42.4)	1.19	0.90	1.56	0.21	1.34	1.01	1.80	0.04	1.35	1.01	1.80	0.040
Unhealthy	325 (19.6)	90 (26.5)	1.63	1.19	2.22	0.002	1.75	1.26	2.44	0.001	1.76	1.25	1.46	0.001

CI: confidence interval; OR: odds ratio; HAC: Harmful Alcohol Consumption. P value of <0.05 were considered statistically significant.

### Temporal validation models

In the temporal validation assessment, the multivariate logistic model exhibited strong discrimination performance, yielding a ROC of 0.78, a sensitivity of 57%, and a specificity of 77%. In the subgroup of HD patients, the model’s discrimination was even more pronounced, achieving a ROC of 0.80, a sensitivity of 72%, and a specificity of 81% (Figure 3). The multivariate random-effects model did not improve the predictive ability of the temporal validation model (likelihood-ratio test: *P*=0.07 with an ROC of 0.76) when applied to the whole cohort, confirming the model’s stability over time regardless of the baseline characteristics of reevaluated patients, and consistent with the MCAR pattern of lost follow-up.

**Figure 3:** – Receiver Operating Characteristic Curve of Heart Disease Patients Associated with Harmful Alcohol Consumption.

### Changes in lifestyle behavior scores are associated to variations in harmful alcohol consumption

Moreover, among patients who underwent two evaluations, an improvement in LBS to a healthier status (observed in 13.2% of the patients) was followed by a reduction in HAC (7.9%, p=0.02) after a three-year follow-up period. The same trend was observed in patients whose LBS deteriorated, with an increase in HAC (3.8% vs. 7.0%, p=0.01).

The central illustration illustrates the main conclusions of the study.

## Discussion

This manuscript introduces a novel LBS model, demonstrating a robust predictive capacity for identifying HAC in the general population, particularly in individuals with HD. The development of this tool emphasizes the importance of categorizing lifestyle behaviors, particularly in the context of HD risk management. Our study’s novel findings align with existing research in the general population, revealing a consistent correlation between unhealthy lifestyle behaviors – such as smoking, lack of physical activity, poor mental health – and higher rates of alcohol abuse.^14-18,26-28^

In our study, we observed that older individuals particularly those with HD had a lower tendency for HAC. The protective effect of aging against HAC in HD patients is stronger than that observed in volunteers (5% vs 4% respectively). This finding suggests a heightened awareness of HD population about the importance of reducing exposure risks.

The proportion of men with HAC tends to be higher than that of women in the general population. Although this trend has decreased over the years, our study found that the risk of HAC remains high among men, both in volunteers and in HD patients.^29^

In our LSB score, we considered BMI as an indicator of dietary patterns. Interestingly, the United States Department of Agriculture (USDA), while not including alcoholic beverages as a component of the dietary pattern, emphasizes the significance of accounting for the calories present in beverages to maintain healthy dietary limits. Failing to adhere to these limits can potentially contribute to an unfavorable lifestyle.^30^

We also considered physical activity as a lifestyle factor with a significant impact on alcohol consumption. A recent study reported a significant increase in non-HAC cases among those with higher fitness levels. In our opinion, these findings are aligned with the concept that non-HAC is part of a healthy lifestyle.^31,32^ Furthermore, tobacco use, which is strongly associated with HAC,^15-18^ was also included in the LBS.

Our study also incorporated psychosocial behavior as a component of the score, recognizing its significant interaction with alcohol consumption and its impact on lifestyle. The relationship between alcohol consumption and stress is complex. Although alcohol can temporarily alleviate anxiety, it can also act as a stressor, increasing the likelihood of HAC in response to stress, often referred to as stress-related excessive alcohol consumption.^27^ Additionally, depression has been associated with HAC, particularly among university students.^28^

In summary, the heterogeneity of the relevant factors mentioned above justifies the development of a scoring system like the one we performed in the general population and in patients with HD.^9^ In practical terms, for this poorly studied population, our score results can drive interventions focused on lifestyle improvement to combat HAC, such as smoking cessation programs, promotion of physical activity, and mental health therapies.

A limitation of this study is the non-inclusion of sleep behavior evaluation in our scoring. Although sleep behavior has been reported as an influential lifestyle factor, its association with HAC still does not have a significant impact compared with the other factors included in our LBS. Another limitation of this study is the lack of formal external validation of the model. However, this potential bias may have been minimized by the temporal validation conducted, which reduces the likelihood of bias in large populations, particularly in homogenous groups like HD patients.^33^

## Conclusions

This study showed that LBS, a new lifestyle score, is a reliable predictor of HAC in HD patients. Also, it was observed that improving lifestyle behavior may help to reduce HAC.

## Supplemental Materials:

Supplemental Material For additional information, please click here.
